# Older Adults With NSTEMI: Picking the Patient’s Future

**DOI:** 10.1016/j.jacadv.2025.102271

**Published:** 2025-10-29

**Authors:** Abdulla A. Damluji, Naila Ijaz, Michael G. Nanna

**Affiliations:** a Cardiovascular Center on Aging, Department of Cardiovascular Medicine, Cleveland Clinic, Cleveland, Ohio, USA; b Division of Cardiology, Department of Medicine, Thomas Jefferson University Hospital, Philadelphia, Pennsylvania, USA; c Division of Cardiology, Department of Medicine, Yale School of Medicine, New Haven, Connecticut, USA

**Keywords:** acute coronary syndrome, aged, conservative treatment, frailty, percutaneous coronary intervention

**Figure:**
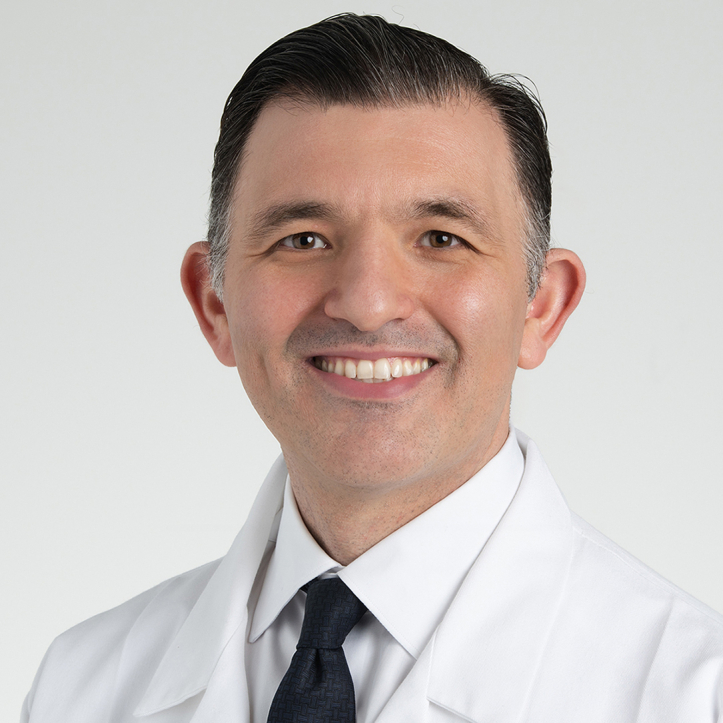

In “The Garden of Forking Paths”, Jorge Luis Borges wrote a short story in England about a Chinese spy for Germany during the First World War. The spy, who is a professor named Yu Tsun, had to deliver a secret message to Germany because he knew he was being pursued. His message is the location of a new British artillery park. He decides that the only way to deliver the message is to kill a man named Stephen Albert, who lives nearby. He knows that after he kills him, his name will be in the newspaper and that will be a signal to his German chief that the artillery park is located in the town of Albert’s residence. Dr Albert was a scholar who studied Yu Tsun’s ancestors and described the world as an infinite branching possibilities and parallel realities. According to Dr Albert, each choice or decision can lead to a different future. That image fits the care for older patients with non-ST-segment elevation myocardial infarction (NSTEMI). One path includes invasive cardiovascular care for management of acute coronary syndrome (ACS), which may prevent future myocardial infarctions (MIs) and urgent revascularization, but it can lead to major bleeding events. The other path includes a conservative approach to management where it avoids procedure-related adverse events, but it increases the risk of future ischemic events later in life. Borges helps us see these decisions as pathways (not a single right answer) that reflect what the future that an older patient prefers.

Idowu et al^1^ pooled data from 8 randomized trials in adults age 70 years or older with NSTEMI. This meta-analysis included 3,275 patients with NSTEMI that were randomized to either an invasive strategy (n = 1,628) or a conservative strategy (n = 1,647). Nearly half of the study population was women and both groups had comparable prevalence of existing coronary artery disease, including patients who had undergone percutaneous coronary intervention (PCI) and coronary artery bypass grafting (CABG) before. They found no difference in all-cause mortality or cardiac death between a routine invasive and a conservative strategy. However, there was a greater risk of myocardial reinfarction and unplanned revascularization in the conservatively treated group, whereas invasive management was associated with a greater risk of major bleeding.^1^

The findings of this meta-analysis align with those of a recently published meta-analysis of 9 trials by Rout et al.^2^, which reported that an invasive strategy for NSTEMI was associated with a significantly reduced risk of the composite outcome of death or MI; however, no significant difference was demonstrated in all-cause mortality, cardiovascular death, or major bleeding. A key distinction which must be recognized when analyzing the results of these 2 meta-analyses is that Rout et al. evaluated a composite endpoint, whereas Idowu et al. analyzed death and MI separately. Consistently, both analyses demonstrated a higher incidence of myocardial reinfarction with conservative management, reinforcing that there is a role for an invasive approach in reducing recurrent ischemic events, although without a survival benefit. Similar findings were reported by Kotanidis et al.^3^ in an individual patient data meta-analysis that showed the risk of MI and unplanned revascularization is lower in older patients with NSTEMI treated with routine invasive strategy compared to conservative strategy. There was no difference in all-cause mortality, cardiovascular death, or stroke between the 2 strategies.^3^

The study by Idowu et al. was conducted with an intention-to-treat approach, hence there was significant crossover between the treatment arms. In the invasive group, revascularization was only performed in a portion, with 46.6% to 61.3% treated with PCI and 1.0% to 5.8% treated with CABG. In the conservative arm, 0% to 48.9% underwent cardiac catheterization with 0% to 22% treated with PCI and 0% to 11.5% treated with CABG. This reflects the complexity in decision-making in this patient population, where careful consideration of anatomy, geriatric syndromes, and comorbidities is required to make decisions regarding whether to proceed with revascularization or choose a more conservative approach. In addition, there was heterogeneity between the trials with regards to follow-up period, which is also likely to impact the mortality and revascularization outcomes.

The follow-up duration ranged from 6 months to 5.3 years. For the trials with limited follow-up periods, reinfarctions and deaths may have been missed, potentially biasing results toward conservative treatment. In addition, as pointed out by the authors, the time to revascularization was variable between the trials, with only a few trials including participants who underwent angiography within 24 hours.^4^ The benefit from revascularization relies on the timing of intervention, with delayed revascularization mitigating treatment effect.^4^

The results of this meta-analysis must be interpreted with caution, recognizing that the care of the older adult is nuanced and cannot be reduced to chronological age alone.^5^ Clinical decisions for older adults with ACS must account for multiple variables beyond age.^6^ An intriguing observation from this meta-analysis that highlights this fact is that adults 80 years and older were less likely to have major bleeding compared to adults 70 to 79 years of age. Although these data have limited clinical applicability, it does raise an important point: some older adults that are “younger” in chronological age may be more frail than those who are older but exhibit signs of robustness.^5^ Thus, treatment strategies should be guided not simply by age, but by a broader assessment that includes frailty, cognitive impairment, multimorbidity, polypharmacy, and quality of life.^7^

The findings of this meta-analysis are consistent with current recommendations from the American Heart Association scientific statement on the management of ACS in older adults, reinforcing that primary PCI in older adults with NSTEMI may reduce recurrent MI and repeat revascularization.^8^ This scientific statement and expert panels from the American College of Cardiology Geriatric Cardiology Leadership Council, recommend involving geriatric cardiology assessment in the multidisciplinary team evaluating older patients undergoing cardiovascular interventions.^6^^,^^9^ In addition, the CRUSADE bleeding score may be used to identify those at risk for major bleeding. However, these scores only offer a starting point. What is urgently needed are tools that reflect the syndromes associated with aging: frailty, cognitive decline, multimorbidity, and polypharmacy.^7^ These factors often determine whether older adults will meaningfully benefit from invasive therapy, yet they remain absent from most clinical algorithms. Until risk scores for older adults are developed that integrate these dimensions, decision-making must involve a multidisciplinary approach that involves geriatricians, family members, and primary care physicians, ensuring that decisions are patient-centered.^7^

Although this meta-analysis contributes valuable evidence to the existing body of literature on management of NSTEMI in older adults, the decision to proceed urgently to the catheterization laboratory remains highly nuanced. Despite the lack of survival benefit, older adults may benefit from invasive strategies as revascularization may improve morbidity, preventing reinfarctions, potentially reducing rehospitalizations and the need for urgent revascularization at another time. However, in those older adults with a poor functional status, comorbidities like cancer, advanced chronic kidney disease, high risk of bleeding, existing frailty, or cognitive impairment, greater care needs to be taken to weigh the risks and benefits. Additional research is needed to develop risk scores that integrate not only ischemic and bleeding risk, but also frailty, cognitive status, and comorbidities, so that clinicians can be better guided in delivering the optimal individualized therapy for each patient.

Borges in his writings reminds us that 1 decision pathway is rarely the right path. The story concludes with Yu Tsun killing Dr Albert, knowing that his name will be tied with the name Albert and the German will successfully bomb the artillery park. His act of murder is both a literal and a metaphorical one. It reflects the infinite possibilities and choices that define our lives, which mirrors our complex and nonlinear nature of a garden with branching trees. Our job as clinicians is to show patients the likely futures on each branch, pick the one that matches their goals and risks, and then proceed with caution particularly for older more frail patients with NSTEMI. There is now more than ever a need for better tools to help us select patients most suitable for revascularization strategies incorporating the syndromes that are encountered at extremes of age. We need more evidence-based interventions in older patients with more pragmatic trials with liberal inclusion criteria. Such evidence will guide the clinical decision-making when combined with patient values and priorities.

## Funding support and author disclosures

Mentored patient-oriented research career development award from the 10.13039/100000050National Heart, Lung, and Blood Institute K23-HL153771. Dr Nanna reports unrelated current research support from the 10.13039/100005485American College of Cardiology Foundation supported by the George F. and Ann Harris Bellows Foundation, the 10.13039/100006093Patient-Centered Outcomes Research Institute (PCORI), the 10.13039/100008479Yale Claude D. Pepper Older Americans Independence Center (P30AG021342), and the 10.13039/100000049National Institute on Aging (K76AG088428) and received personal fees from Heartflow, Inc, Merck, and Novo Nordisk. Dr Damluji receives research funding from the Pepper Scholars Program of the Johns Hopkins University Claude D. Pepper Older Americans Independence Center funded by the 10.13039/100000049National Institute on Aging P30-AG021334; mentored patient-oriented research career development award from the 10.13039/100000050National Heart, Lung, and Blood Institute K23-HL153771; the 10.13039/100000049NIH National Institute of Aging R01-AG078153; and the 10.13039/100006093Patient-Centered Outcomes Research Institute (PCORI). All other authors have reported that they have no relationships relevant to the contents of this paper to disclose.
